# Hippocampal and limbic microstructure changes associated with stress across the lifespan: a UK biobank study

**DOI:** 10.1038/s41598-024-71965-4

**Published:** 2024-09-17

**Authors:** Elizabeth McManus, Hamied Haroon, Niall W. Duncan, Rebecca Elliott, Nils Muhlert

**Affiliations:** 1 School of Health Sciences, The University of Manchester, H.18 Coupland 1 Building, Oxford Rd, Manchester, M13 9PL UK; 2 Graduate Institute of Mind, Brain and Consciousness, Taipei Medical University, Taipei, Taiwan; 3 Brain and Consciousness Research Centre, TMU Shuang Ho Hospital, New Taipei City, Taiwan

**Keywords:** Childhood stress, Adulthood stress, Diffusion MRI, Sex differences, Microstructure, Lifespan, Cognitive neuroscience, Risk factors

## Abstract

Experiencing highly stressful events can have detrimental and lasting effects on brain morphology. The current study explores the effects of stress during childhood and adulthood on grey matter macro- and microstructure using a sub-sample of 720 participants from the UK Biobank with very high or very low childhood and adulthood stress scores. We used T1-weighted and diffusion MRI data to assess grey matter macro- and microstructure within bilateral hippocampus, amygdala and thalamus. Findings showed that childhood stress is associated with changes in microstructural measures bilaterally within the hippocampus and amygdala. No effects of adulthood stress on brain microstructure were found. No interaction effects between sex and stress (either childhood or adulthood) were observed for any brain imaging measure. Analysis of sub-segments of the hippocampus showed that childhood stress predominantly impacted the bilateral heads of the hippocampus. Overall, these findings suggest that highly stressful experiences during childhood, but not adulthood, have lasting impact on brain microstructure. The effects of these experiences in childhood appear to persist regardless of experiences of high or low stress in adulthood.

## Introduction

Stress is common in daily life and may occur at any point across the lifespan, but repeated exposure to highly stressful or traumatic experiences may have long lasting impacts decades later on both mental and physical health^1–3^. One of a number of biological processes that occurs as a result of stress is the activation of the hypothalamic–pituitary–adrenal (HPA) axis, culminating in the release of cortisol^4^. Although beneficial in the short term, prolonged overexposure to stressors and sustained high levels of circulating cortisol can exert damage on areas of the brain that are rich in cortisol receptors (e.g. the limbic system) and are also critically involved in regulating the HPA axis^5,6^. Subsequent disruption in HPA axis functioning can negatively impact metabolic and immune systems^7^. This damage to the areas of the limbic system highlights a mechanism through which high levels of stress may negatively impact both mental and physical health.

Stress throughout the lifespan has been linked to alterations in brain structure. During childhood, experiences of highly stressful situations (such as abuse or deprivation) have been shown to be associated with changes in grey matter volume, particularly in regions within the limbic system including the hippocampus, amygdala and thalamus^8–11^ and changes in white matter microstructure in the corpus callosum and uncinate fasciculus^8,12–14^. Similar changes in macro- and microstructures of the hippocampus, amygdala and thalamus are seen in adults with a history of exposure to high levels of stress or trauma^15–22^. This is in line with the ‘glucocorticoid-cascade hypothesis of stress’ posited by Sapolsky and colleagues, which details the heightened sensitivity of the limbic regions to glucocorticoids^23–25^.

The potential impact of highly stressful events on brain structure as a result of heightened activation of the HPA axis, may differ between males and females. Although there are some discrepancies, it is generally reported that for healthy adults, males experience greater cortisol increases due to stress than females^26,27^. However, this effect is not necessarily the same across the whole lifespan. When considering teenagers going through puberty, HPA axis reactivity has been shown to be increased in females relative to males^28^. A meta-analysis of HPA reactivity differences in under 18’s suggested that although girls have more variable diurnal rhythm and generally higher cortisol reactivity to social stressors than boys, this was not true for all types of stress^29^. Similarly, it has been shown that older (post-menopausal) women show greater cortisol response than age matched males after a cognitive challenge^30,31^. It is also suggested that older women experience higher levels of 24 h cortisol than younger women and younger and older men^31^. Since cortisol levels vary across the lifespan and between sexes, heightened activation of stress response systems, such as the HPA axis, may have different effects on the brain depending on the age at which stressful experiences occurred and the sex of the individual.

The specific impact of stress for males and females on brain macro- and microstructure at different points in the lifespan has been previously demonstrated. For example, girls that have experienced high levels of childhood stress demonstrate significant associations between FA in white matter regions adjacent to the left thalamus, right anterior cingulate and right superior frontal gyrus^32^. Other studies have shown that childhood abuse led to grey matter volume reduction in the hippocampus for males but not females^33^. Many of these studies, however, only explore the impact of stress at either one time point, (e.g. childhood or adulthood, but not both) or in one sex, making it more difficult to directly compare the differences in effects in the brain. Together however, this research highlights the possibility that at different stages in the lifespan, experiencing very high levels of stress may lead to distinct alterations in brain structure and this may also differ between males and females.

While many studies demonstrate alterations in hippocampal structure in relation to both childhood and adulthood stress, there exists some variability in findings between studies^5^. This may in part be due to differences in the impact of stress on specific sub-regions of the hippocampus, rather than the hippocampus as a whole^34^. There is evidence to suggest that the head of the hippocampus may be more susceptible to structural changes due to stress than other sub-regions^35^. As such, it may also be important to investigate stress related alterations of sub-regions of the hippocampus, in addition to the hippocampus as a whole.

Previously our work has explored the impact of high and low levels of adulthood and childhood stress on white matter microstructure using UK Biobank imaging derived phenotype (IDP) values. We demonstrated that in females, childhood stress was associated with reduced connectivity in white matter structures such as the posterior thalamic radiation and the cingulum of the hippocampus, but in males reductions in connectivity were shown for those who experienced high levels of adulthood, but not childhood, stress^36^. However, in this study, unlike other literature, we observed no grey matter volume IDP differences in the limbic regions of interest^36^. Therefore, in the current study, we hope to further investigate potential grey matter changes, by assessing more subtle microstructural differences in the grey matter that could be suggested to be a precursor to macrostructure volume changes.

Using raw imaging data from the UK Biobank, the current study aims to examine the impact of highly stressful experiences during childhood and adulthood on grey matter macro- and microstructure, and assess if these effects are influenced by sex. We predicted that those experiencing high levels of childhood and/or adulthood stress would show some small reductions in grey matter volume in the hippocampus, amygdala and thalamus. Additionally, we hypothesized that grey matter microstructural measures in these limbic regions would be altered in those experiencing high levels of childhood and/or adulthood stress, reflecting possible decreased microstructural integrity. As microstructural changes have been shown to pre-date macrostructural changes, we predict greater changes in grey matter microstructure. Finally, we anticipate these changes to differ between males and females.

## Methods

### Participants and selecting stress groups

Participant datasets used within the current study were obtained from the UK Biobank’s data release in February 2020 (https://www.ukbiobank.ac.uk/). The UK Biobank is a large-scale health and biomedical study conducted across Great Britain, collecting data from approximately 500,000 volunteers aged 40–69 years old between 2006 and 2010. A subset of these volunteers underwent extensive imaging procedures, including brain MRI (at time of data release *N* = 40,681). Ethical approval was granted to the UK Biobank by the North West Multi-Centre Research Ethics Committee (REC reference 11/NW/0382) and all participants provided informed consent to participate and for their anonymised data to be used. The current study was conducted under approved UK Biobank application number 49224.

Before selecting participants to be included in our analysis, we first removed any participant reporting neurological conditions at the time of the neuroimaging session. While completing the initial UK Biobank assessment, participants also completed several online follow-up questionnaires. From these questionnaires we estimated participants’ levels of childhood stress using five specific questions (Field IDs 20,489–20,491: see Appendix A), and adulthood stress using a further five questions (Field IDs 20,521–20,525: see Appendix B). All questions were scored on the same 5-point rating scale, and reverse coded where necessary. Participants with missing data for these questionnaires were excluded leaving *N* = 147,131. Scores for each childhood stress and adulthood stress question were then summed to give a total childhood stress and a total adulthood stress score.

Based on overall scores of childhood stress and adulthood stress, high/low childhood and adulthood stress groups were then defined. Due to the large majority of participants scoring the lowest possible score (0) for both childhood stress and adulthood stress, the criteria for low stress was set as a score of 0 (no experience of childhood or adulthood stress). The questions used to define childhood stress were taken from the CTQ^37^ and given that there is no set criteria within the CTQ to define “high levels” of trauma/stress, our a priori criteria for categorisation to the high childhood and adulthood stress was set to 2 standard deviations above the mean (high childhood stress > 6.55, high adulthood stress > 7.05).

**Fig. 1:**
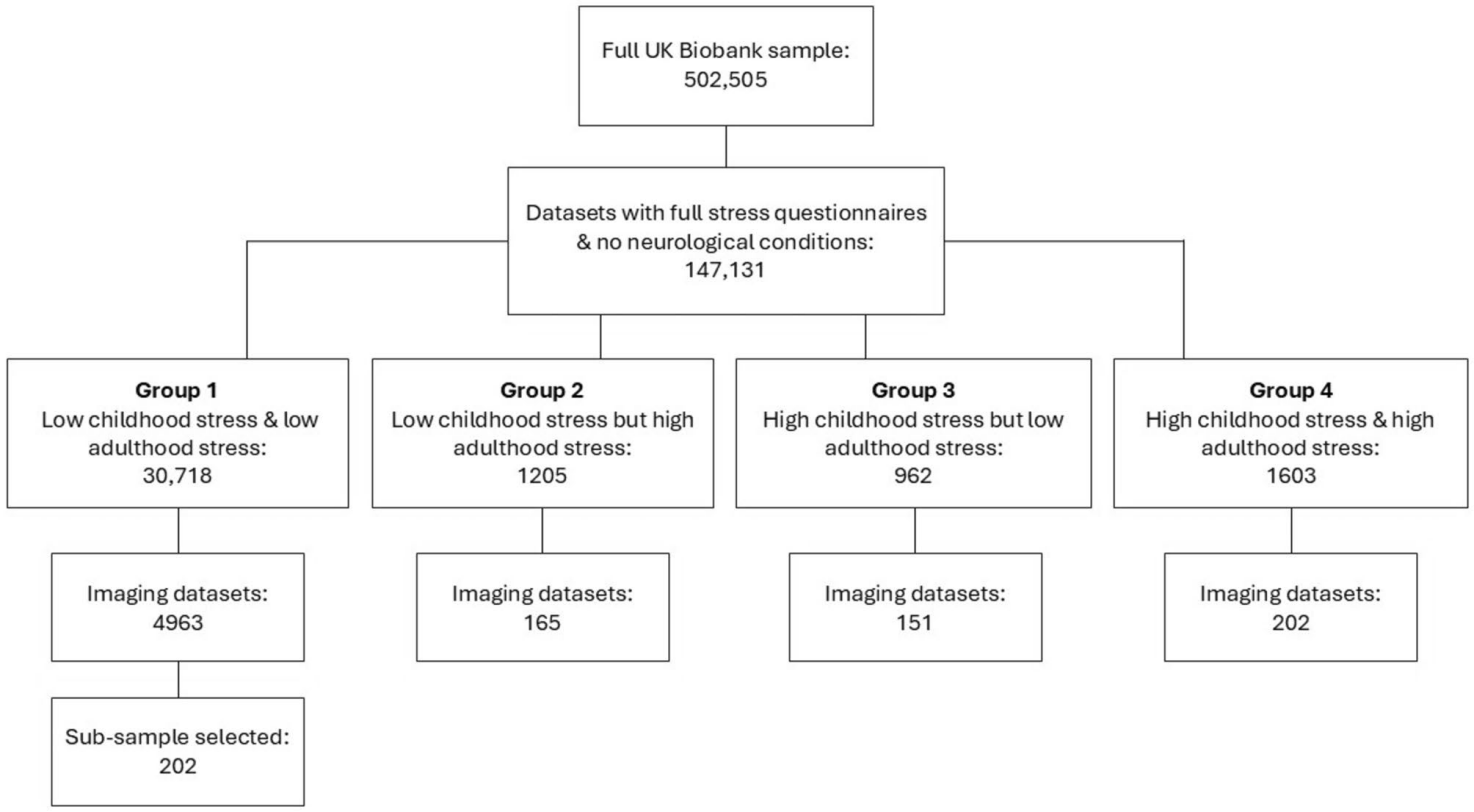
Flow chart demonstrating sample size at each stage of group selection.

Using these criteria, we selected participants for inclusion in this analysis on the basis of four defined stress groups: (1) low childhood stress and adulthood stress (LC/LA), (2) low childhood stress but high adulthood stress (LC/HA), (3) high childhood stress but low adulthood stress (HC/LA) and (4) high childhood stress and adulthood stress (HC/HA). Participants with scores not meeting this criteria were removed from the sample. Additionally, participants without neuroimaging data were also removed at this point, leaving 5481 meeting the stress criteria with neuroimaging data. As there were substantially more participants in the LC/LA than any other groups, we randomly sampled from the LC/LA group to create a sub-sample approximately the same size as other stress groups (N = 202). Figure 1 shows a flow chart of how group sizes were determined and how this led to our final sample size *N* = 720.

### Brain image acquisition and processing

The UK Biobank used three dedicated imaging centres, each equipped with identical MRI scanners (3.0 T Siemens Skyra, software VD13), and used the same standard Siemens 32-channel receiver head coil. Details of the MRI protocol and processing steps have previously been reported in full^38,39^. For the current study, we used the pre-processed and quality checked NIFTI images from the UK Biobank imaging team^39^.

#### Regional volume analyses

**Fig. 2:**
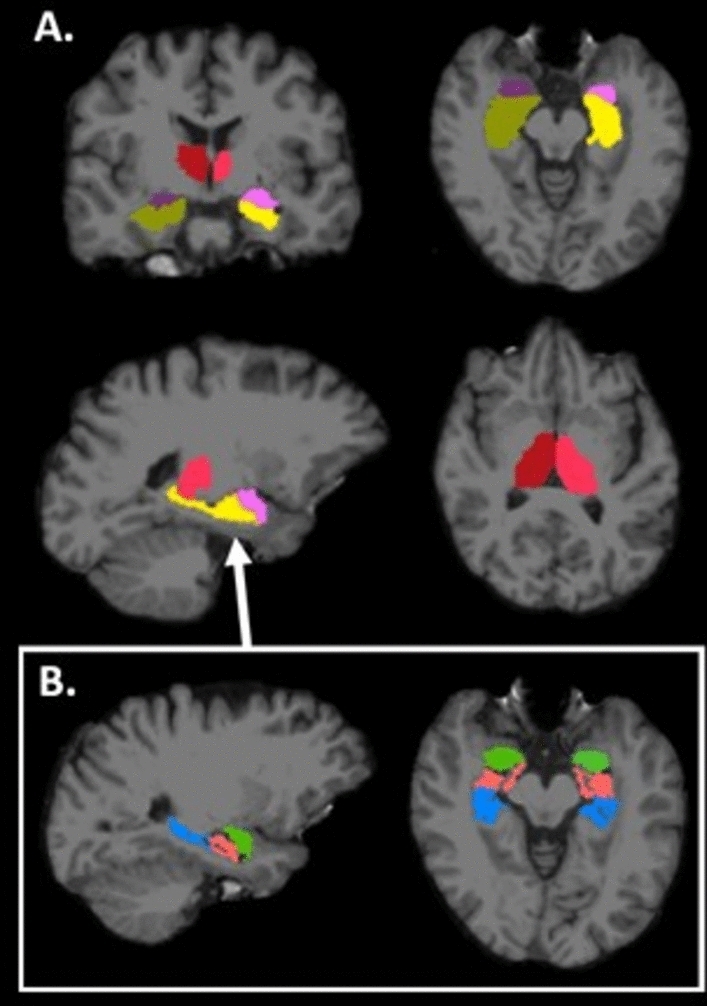
(**A**) Region maps for grey matter regions of interest: amygdala (left in dark purple and right in light pink), hippocampus (left in dark yellow and right in light yellow) and thalamus (left in dark red and right in lighter red). (**B**) Region maps for Hippocampus segmented into head (green), body (light pink) and tail (blue) regions.

To compare regional volumes, we used pre-processed raw images provided by the UK Biobank neuroimaging team^39^. Cortical reconstruction and volume segmentation of these images was performed using FreeSurfer v7.1.1 image analysis software documented online (http://surfer.nmr.mgh.harvard.edu/). The technical details of this process have been described in depth in previous publications^40,41^, but briefly included removal of non-brain tissue, transformation into standard space, segmentation into grey matter (cortical and deep subcortical), white matter and CSF in addition to segmentation into specific regional structures. The specific grey matter regions of interest for this study were bilateral thalamus, hippocampus, and amygdala (see Fig. 2A).

#### Microstructural analyses

Several diffusion metrics were used to measure microstructural properties within the regions of interest for this study. Diffusion tensor imaging (DTI) measures included mean diffusivity (MD), fractional anisotropy (FA), axial diffusivity (AD), and radial diffusivity (RD). We also used diffusion measures more suited for analysing grey matter regions: Neurite orientation dispersion and density imaging (NODDI,^42^). The NODDI measures included were intracellular volume fraction (ICVF), isotropic volume fraction (ISOVF) and neurite orientation dispersion (OD). Diffusion orientation complexity (DOC) analyses was also used to estimate of the probability of having 1, 2, 3, or more than 3 dominant diffusion orientations (donated as P(n = 1, 2, 3 or > 3) respectively). DOC has previously been shown to be sensitive to neurodegeneration in grey matter^43–45^. FSL’s FLIRT (https://fsl.fmrib.ox.ac.uk/fsl/fslwiki;^46^ was used to linearly register diffusion maps to the FreeSurfer outputs in the same participant.

These diffusion analyses were conducted for the three grey matter regions of interest: the thalamus, hippocampus and amygdala (see Figs. 2A). To further investigate the microstructural properties of the hippocampus, this region was later sub segmented bilaterally into head, body, and tail subfields (see Fig. 2B) using FreeSurfer tools^47^ and diffusion analyses were run for these subfields.

### Statistical analysis

The handling of raw UK Biobank data, creation of sub samples and groups and the statistical analysis for all comparisons was conducted using R^48^. Given the large sample sizes, all imaging measures were assessed for normality using Kolmogorov–Smirnov (KS) tests. Any data points three or more standard deviations away from the mean were considered outliers and removed from the relevant analyses. Kruskal–Wallis tests were used to compare ages between male and female participants and between stress groups to assess for potential differences between samples.

Although data was selected for inclusion in this study on the basis of the four high/low childhood/adulthood stress groups, to allow for the assessments of interactions between childhood and adulthood stress, a 2 (childhood stress: high and low) × 2 (adulthood stress: high and low) × 2 (sex: male and female) ANCOVA model was run, with age as the covariate. This ANCOVA model was used to assess the main effects of childhood stress, adulthood stress, sex and the interactions between these variables on volume and diffusion measures for the regions of interest included in this study. As multiple regions of interest and multiple imaging measures were compared in each of our analyses, false discovery rate (FDR) multiple comparisons corrections were used to adjust significance values (*p*-values set to < 0.05). Only comparisons surviving multiple corrections were reported as significant. Partial eta squared effect sizes were calculated and reported.

## Results

### Group differences: age and sex

An independent sample *t*-test showed a significant overall age difference (*t*(354.1) = -3.43, *p* < 0.001) within the sample, with males (mean = 55.22 years, SD = 7.74 years) being slightly older than females (mean = 53.06 years, SD = 7.31 years). A Kruskal–Wallis rank sum test revealed no differences in age between low and high stress groups in both the male (*χ*^*2*^^3^ = 4.89, *p* = 0.18) and female (*χ*^*2*^(3) = 2.31, *p* = 0.51) sample. Mean ages per group are shown in Table 1 and mean scores for childhood and adulthood stress from each group are shown in Table 2.

**Table 1:** Male, female and overall samples number of participants and mean ages (with standard deviation in parentheses) for each stress group.

	Males		Females		Overall	
	*N*	Age	*N*	Age	*N*	Age
Group 1 (low childhood stress and low adulthood stress)	84	55.29 (7.64)	118	53.94 (7.31)	202	54.5 (7.46)
Group 2 (low childhood stress and high adulthood stress)	34	55.74 (7.70)	131	52.98 (7.11)	165	53.54 (7.30)
Group 3 (high childhood stress and low adulthood stress)	55	56.31 (7.68)	95	52.75 (7.90)	150	54.05 (7.98)
Group 4 (high childhood stress and high adulthood stress)	31	52.55 (7.94)	171	52.68 (7.13)	202	52.66 (7.24)

**Table 2:** Mean and standard deviations (below in parentheses) for childhood and adulthood stress scores for each of the high and low stress groups.

	Childhood stress	Adulthood stress
Group 1 (low childhood stress and low adulthood stress)	0 (0)	0 (0)
Group 2 (low childhood stress and high adulthood stress)	0 (0)	9.23 (1.68)
Group 3 (high childhood stress and low adulthood stress)	8.73 (2.17)	0 (0)
Group 4 (high childhood stress and high adulthood stress)	10.23 (3.08)	10.26 (2.70)

### Impact of stress on grey matter volume

Differences in volume of the left and right hippocampus, amygdala, and thalamus were assessed using 2 (childhood stress) × 2 (adulthood stress) × 2 (sex) ANCOVA model. Age was a significant covariate in all regions. No main effects of childhood or adulthood stress were observed in any regions. Main effects of sex were observed in grey matter volume of the left and right hippocampus and the left thalamus. No two or three way interactions were observed between sex, childhood stress or adulthood stress.

### Impact of stress on grey matter microstructure

**Fig. 3:**
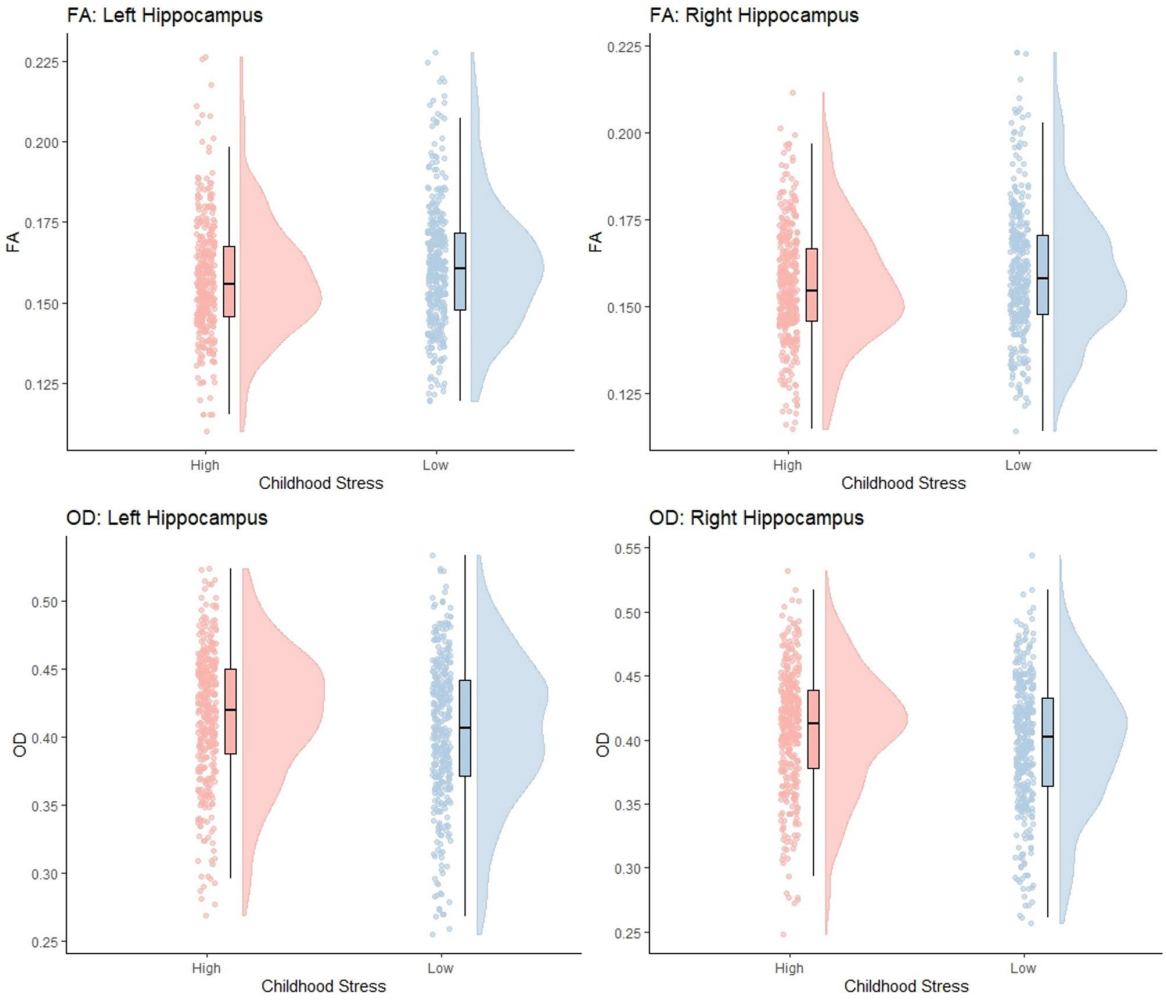
Raincloud plot representing data distributions for values of FA and OD values in the bilateral hippocampus between those with high and low levels of childhood stress. Box plots represent median and interquartile ranges of these values.

Microstructural changes in the bilateral hippocampus, amygdala and thalamus were assessed using the same 2 × 2 × 2 ANOCVA model. Significant main effects of childhood stress were shown for several measures of diffusion bilaterally in the hippocampus and amygdala. No significant effects of childhood stress are seen within the thalamus. All significant effects are reported in Table 3. As shown in Fig. 3, high level of childhood stress led to decreases in FA and increases in OD, bilaterally in the hippocampus. Similarly, OD was increased in the left amygdala in those with high levels of childhood stress relative to those with no history of childhood stress. In the left amygdala and the left and right hippocampus, P(n = 3) was increased in the high childhood stress group, as was P(n > 3) in the left amygdala and right hippocampus. Finally, P(n = 1) was decreased in the right hippocampus and left and right amygdala for those experiencing high levels of childhood stress.

**Table 3:** Statistics for all significant the main effects of childhood stress, and the associated partial eta squared effect sizes, for diffusion measures in bilateral hippocampus and amygdala.

Diffusion measure	Region	F	*p*	*n*_*p*_^*2*^
FA	Left hippocampus	12.05	0.016	0.02
	Right hippocampus	11.89	0.016	0.02
OD	Left hippocampus	10.27	0.016	0.01
	Right hippocampus	10.41	0.016	0.01
	Right amygdala	5.50	0.016	0.008
P (n = 1)	Right hippocampus	7.88	0.031	0.01
	Left amygdala	9.66	0.016	0.01
	Right amygdala	8.15	0.031	0.01
P (n = 3)	Left hippocampus	7.21	0.038	0.01
	Right hippocampus	110.01	0.016	0.01
	Left amygdala	7.85	0.031	0.01
P (n > 3)	Right hippocampus	7.22	0.038	0.01
	Left amygdala	9.68	0.016	0.01

Reported p-values are FDR adjusted.

Within these significant findings, a significant main effect of sex was also seen within the analysis of OD in the left hippocampus, P(n = 1) in the left and right amygdala, P(n = 3) left and right hippocampus, and P(n > 3) in the left amygdala. Similarly, age was a significant covariate in most but not all regions. Age was not a significant covariate in analysis of OD in bilateral hippocampus, or for P(n = 1) and P(n = 3) measures in the right hippocampus.

No main effects of adulthood stress were observed in any of the regions of interest for any measure of diffusion. Similarly no significant 2 or 3 way interactions were observed between childhood stress, adulthood stress and sex for any measure in any region of interest.

### Hippocampal subsegments

**Fig. 4:**
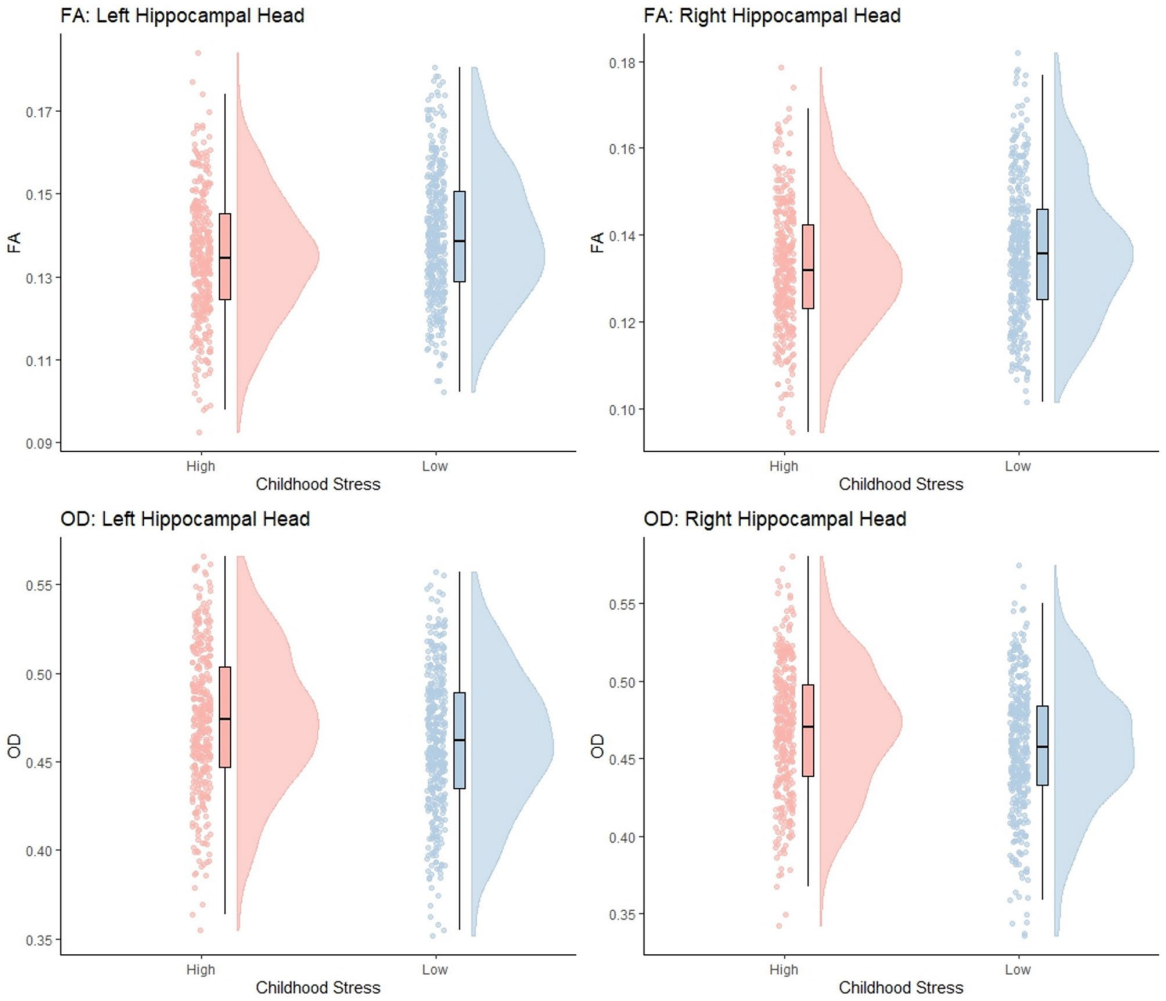
Raincloud plots representing data distributions for values of FA and OD values in the bilateral heads of the hippocampus between those with high and low levels of childhood stress. Box plots represent median and interquartile ranges of these values.

After segmenting the hippocampus into head, body and tail segments, all measures of diffusion were again compared using the same 2 × 2 × 2 ANOCVA model. Much like the previous analysis, significant main effects for childhood stress were observed for several diffusion measures in the bilateral hippocampal heads and the right body of the hippocampus. All significant effects of childhood stress on diffusion measures within subsegments of the hippocampus are shown in Table 4. No significant effects of childhood stress were shown in the hippocampal tail. As shown in Fig. 4, those with high childhood stress again showed decreased FA and increased OD in the bilateral hippocampal heads. This pattern is also shown in the right body of the hippocampus. Increases in RD were also shown for high childhood stress in the left and right hippocampal heads. Additionally, DOC measures also showed increased in P(n = 3) and P(n > 3) and decreases in P(n = 1) in the left and right hippocampal heads and the right body of the hippocampus for those experiencing high childhood stress.

**Table 4:** Statistics for all significant the main effects of childhood stress, and the associated partial eta squared effect sizes, for diffusion measures in bilateral subsegments of the hippocampus.

Diffusion measure	Hippocampal subsegment	F	*p*	*n*_*p*_^*2*^
FA	Left head	22.69	< 0.001	0.03
	Right head	14.55	0.002	0.02
	Right body	8.87	0.020	0.01
OD	Left head	20.34	< 0.001	0.03
	Right head	15.88	0.001	0.02
	Right body	7.43	0.029	0.01
RD	Left head	10.64	0.008	0.01
	Right head	7.63	0.029	0.01
P(n = 1)	Left head	15.56	0.002	0.02
	Right head	7.72	0.029	0.01
	Right body	6.50	0.043	0.01
P(n = 3)	Left head	15.53	0.001	0.02
	Right head	10.98	0.008	0.02
	Right body	6.95	0.035	0.01
P(n > 3)	Left head	12.4	0.004	0.02
	Right head	7.40	0.029	0.01
	Right body	7.78	0.029	0.01

Reported p-values are FDR adjusted.

Within these regions showing main effects of childhood stress, age was a significant covariate when comparing P(n = 3) and P(n > 3) in the right body of the hippocampus, FA and RD in the left and right hippocampal heads, and OD in the left hippocampal head. Main effects of sex were seen for analysis of P(n = 3) and P(n > 3) in the left and right hippocampal heads.

No main effects of adulthood stress were found when analysing any measure of diffusion in any of the hippocampal subsegments. Similarly no significant 2 or 3 way interactions were observed between childhood stress, adulthood stress and sex for any measure in any subsegment.

## Discussion

The findings of the current study suggest that high levels of stress, only during childhood, are associated with decreased microstructural integrity in the hippocampus and the amygdala. Critically, we did not see this effect for adulthood stress, nor did we see any interaction effects between childhood and adulthood stress in any regions for any diffusion measures. These findings were somewhat in line with both our hypotheses and the previous literature, suggesting that high levels of stress during childhood have negative impacts on the structure and function of the brain^8,9^. Unlike some previous literature, we did not see volume reductions^8,9^, but instead observed more subtle changes in the grey-matter microstructure of these regions.

Childhood stress was linked to decreased microstructural integrity as evidenced by changes in diffusion measures within the bilateral hippocampus and amygdala. More specifically, when compared to those with no history of childhood stress, those experiencing high childhood stress showed decreases in FA, increases in OD, and decreased P(n = 1), but increased P(n = 3) and P(n > 3). Similar findings of high childhood stress being linked to altered hippocampal structure have been shown in previous literature^8,49^. Further analysis of hippocampal segmentations revealed that these findings appear to be driven by changes in microstructure in the head of the left and right hippocampus, and the body of the right hippocampus. This finding is in line with previous work that has shown that people struggling with post-traumatic stress disorder (PTSD) show alterations specifically in the head of the hippocampus^35^. Taken together, the findings from this study and similar findings in previous literature may indicate that early stressful experiences have disproportionately larger effects on grey matter microstructure of the amygdala and hippocampus, particularly the head of the hippocampus.

Contrary to our initial hypothesis however, we observed no impact of highly stressful experiences during adulthood for any volume or diffusion measures. This finding differs from previous studies that have shown alterations in brain macro- and microstructures in people with a history of exposure to extremely high levels of stress during adulthood^15–19^. One key difference between this study and the previous literature is that many of the previous studies have explored the impact of stress on the brain within clinical samples struggling with stress related mental health conditions (e.g. PTSD and depression), whereas the current study took participants from a general population dataset and as such includes participants both with and without these diagnoses. One potentially important factor not measured in this study that could underpin the differences in brain volume changes due to stress could be individuals’ resilience when dealing with these situations. Resilience, or being able to cope with these highly stressful experiences, could be preventative both in terms of developing mental health conditions and the negative impact on the brain.

Additionally, no interactions were observed between sex and either childhood or adulthood stress. This suggests that the impact of stress on brain microstructure within the limbic regions of interest did not differ between males and females. This was somewhat unexpected given the well documented sex differences in response to stress^26,27^and the previous literature that suggests that a history of highly stressful childhood experiences (e.g. neglect and abuse) is associated with reduced hippocampal volume in males but not in females^33^. A possible factor that could contribute to this difference in findings, particularly for childhood stress, may be the exact age at which people were exposed to high levels of stress. The current study is not able to identify at what age these experiences occurred. Therefore, it may be that age at exposure to stressful experiences in childhood, particularly if close to puberty, may be key to demonstrating stress related sex differences in the brain.

Although providing a well powered insight into the impact of stress during childhood and adulthood, this study is not without limitations. This study explored the impact of stressful experiences on the macro- and microstructure of three pre-defined regions of interest in the limbic system. Other brain regions, including the cingulate cortex, striatum and prefrontal cortex, are also related to stress processing and response^50–52^. As such, including these regions of interest within our analysis or conducting a whole brain analysis approach, may have revealed additional alterations in brain structure related to highly stressful experiences during childhood.

Within this study, we are also limited by our definition of childhood and adulthood stress. The UK Biobank used a subset of 5 questions from the CTQ^37^, an approved and widely used method of childhood trauma. A similar set of questions is also used to assess similar forms of stress, trauma and abuse experienced during adulthood; however, these questions do not come from a formal measure of adulthood stress. While these questions may not be a perfect measure of all stressful experiences, we believe that by defining distinct high and low stress groups, we were able to differentiate between those experiencing very low and very high levels of stress at various points in the lifespan. Alternatively, selecting participants from a more continuous range of stress scores could allow for the investigation of the impact of stress severity. However, given the limitations with the measures of stress and the absence of a measure of the impact of each experience on the individual, we chose instead to maximise potential differences by comparing just those with very high and very low experience stress.

As noted previously, the measures of stress used within this study lack information about the age at which stress occurred, what impact that experience had on the individual, and how well they coped. With no further detail regarding the exact timing of childhood and adulthood stress, we cannot identify if these events occurred before or during puberty for childhood stress, or before, during, or after menopause in adulthood. Similarly, without knowing how the experience impacted the individual, it must be assumed that these experiences impacted everyone in the same way and for the same length of time. Additionally, given our sample (reflecting the UK Biobank cohort) was predominantly white European, these findings may also not reflect ethnic or cultural variations in the experience of, and ability to cope with, highly stressful life experiences. As such, these findings may not account for the potential influence of developmental hormonal fluctuations or possible individual and cultural differences when coping with stress.

The current study explores the impact of highly stressful experiences during childhood and adulthood on brain macro- and microstructure within regions of interest in the limbic system. Our findings revealed significant changes in diffusion MRI-derived metrics of grey matter microstructure, indicating decreased microstructural integrity, for those experiencing very high levels of stress during childhood compared to those who experienced very low levels of this stress. Changes in diffusion measures were observed bilaterally in the amygdala and the hippocampus, particularly in the left and right head and the right body of the hippocampus. No such changes were observed for those experiencing highly stressful events in adulthood and there were no interactions between either form of stress and sex. These results demonstrate the lasting impact of childhood stress on the brain for both men and women, in a non-clinical sample of 40–69 year old participants. Understanding the lasting impact of these experiences may be beneficial when outlining the need for support and interventions after exposure to stressful experiences during childhood.

## Supplementary Information

Supplementary Information.

## Data Availability

All data use for this study is publicly available from the UK Biobank (to those registered) https://www.ukbiobank.ac.uk/.
