# Supplementary material for: Hippocampal and limbic microstructure changes associated with stress across the lifespan: a UK biobank study
Source: Sci Rep. 2024 Sep 17;14:21735. doi: 10.1038/s41598-024-71965-4 (PMC11408494; doi:10.1038/s41598-024-71965-4)
Supplement: Supplementary file 1 — Supplementary Information. [file 41598_2024_71965_MOESM1_ESM.docx]

**Supplementary Materials**

**Appendix A**

Items used to create child stress scores. Category 145: Online follow up- Mental health- Traumatic events. Field IDs, child stress statement, use of reverse coding and number of each response to the question.

| **Field ID** | **Question/Statement** | **Reverse Coding Used?** | **Prefer not to answer** | **Never True (0)** | **Rarely True (1)** | **Sometimes True (2)** | **Often True (3)** | **Very Often True (4)** |
| --- | --- | --- | --- | --- | --- | --- | --- | --- |
| 20487 | Felt hated by a family member as a child | No | 454 | 132,346 | 9540 | 10,432 | 2502 | 2074 |
| 20488 | Physically abused by family as a child | No | 358 | 127,194 | 16,844 | 10,614 | 1424 | 914 |
| 20489 | Felt loved as a child | Yes | 628 | 2311 | 7325 | 25,660 | 39,848 | 81,576 |
| 20490 | Sexually molested as a child | No | 1852 | 141,849 | 7186 | 5002 | 797 | 662 |
| 20491 | Someone to take to doctor when needed as a child | Yes | 1074 | 3378 | 1305 | 4322 | 16,722 | 130,547 |

**Appendix B**

Two sets of statements and response keys used to create adult stress scores. Category 145: Online follow up- Mental health- Traumatic events. Field IDs, adult stress statement, use of reverse coding and number of each response to the question.

| **Field ID** | **Question/Statement** | **Reverse Coding Used?** | **Prefer not to answer** | **Never True (0)** | **Rarely True (1)** | **Sometimes True (2)** | **Often True (3)** | **Very Often True (4)** |
| --- | --- | --- | --- | --- | --- | --- | --- | --- |
| 20521 | Belittlement by partner or ex-partner as an adult | No | 485 | 119,234 | 14,964 | 15,724 | 3669 | 3272 |
| 20522 | Been in a confiding relationship as an adult | Yes | 4127 | 14,051 | 7401 | 27,692 | 26,848 | 77,229 |
| 20523 | Physical violence by a partner or ex-partner as an adult | No | 491 | 136,776 | 9877 | 7408 | 1291 | 1505 |
| 20524 | Sexual interference by a partner or ex-partner without consent as an adult | No | 515 | 147,762 | 5028 | 3035 | 478 | 530 |
| 20525 | Able to pay rent/mortgage as an adult | Yes | 2448 | 5628 | 1109 | 4031 | 11969 | 132,163 |
